# An alginate–PHMB–AgNPs based wound dressing polyamide nanocomposite with improved antibacterial and hemostatic properties

**DOI:** 10.1007/s10856-020-06484-5

**Published:** 2021-01-20

**Authors:** Laleh Asadi, Javad Mokhtari, Marjan Abbasi

**Affiliations:** Department of Textile Engineering, Faculty of Engineering, University of Guilan, Rasht, 41635-3756 Iran

## Abstract

Wound dressing should be impenetrable against microorganisms and it should keep the wound wet. Gauze and polyamide (PA) substrate were treated with various concentrations of AgNPs (25, 50, 75, and 100 ppm), PHMB (0.2, 0.4, 0.6, 0.8, and 1% w/v), and constant concentration of alginate (0.5% W/V) using a simple dipping method. Prepared samples were characterized by various techniques including Fourier transform infrared spectroscopy and scanning electron microscopy. The results indicated that the particles were successfully applied onto both substrates with an average diameter of particle size of 78 nm on gauze and 172 nm on the PA substrate surface (based on 50 nanoparticles). Antibacterial activity of the prepared nanocomposite against *Staphylococcus* aureus (gram-positive) bacteria on PA substrate and gauze were evaluated using the disc diffusion method. The results indicated that the prepared nanocomposites offer favorable antibacterial properties and bacteria would not grow in culture media. The water uptake capacity test of the treated samples was assessed and the data demonstrated that the water absorption rate significantly increases on both treated substrates (gauze and PA substrate) due to the presence of alginate polymer. Also, observing the results of the coagulation test showed that treated samples caused blood clots on the dressing. This is due to the presence of alginate polymer. The present work demonstrates that the prepared samples offer excellent antibacterial properties and good water uptake capacity that capable of being a potential candidate for wound dressings. Due to the results, the produced PA substrate could be an appropriate replacement for the cotton gauze as a wound dressing.

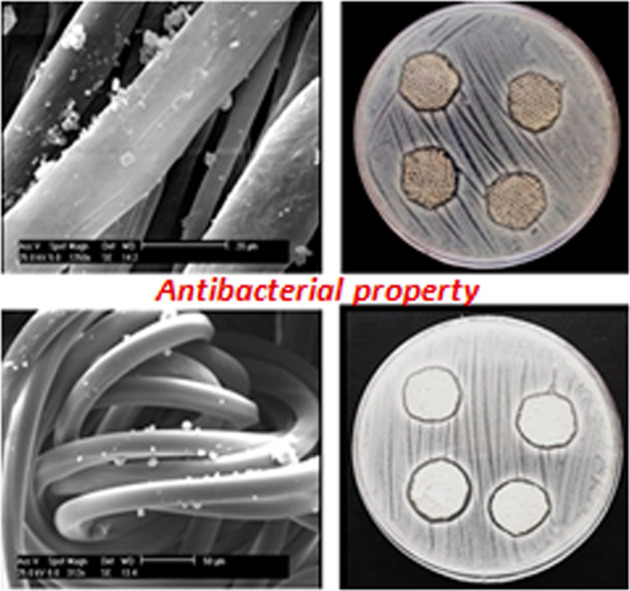

## Introduction

Wound dressings have an important role in the wound care process and are used over various types of wounds and burns for the regeneration and repairing of damaged tissues and also to promote healing and reduce the risk of infection. The ideal wound dressing should reduce healing time and pain, improve regeneration and repairing of damaged tissues, absorb wound extra exudate, promote healing, and also protect the wound against infection [1, 3]. Special dressing for the treatment of wounds has been a prominent goal for scientists for many years. In the past, traditional dressings (e.g., honey, plant fibers, and animal fat) were used to protect the wound against entry the microorganism and keeping the wound dry. Nowadays, some biopolymers and techniques offer dressings with improved properties such as emollient, antioxidant, epithelializing, anti-inflammatory, and antibacterial properties that enhance the wound healing process. Wound dressings should create a moist environment around the wound; cellular guidance, pass moisture vapors, and low bacterial load [3, 4].

Both natural and synthetic polymers are used for wounds and burns dressing. Among them, some polysaccharides (i.e., *β*-glucans, dextran, hyaluronic acid, chitosan, heparin, and alginate) are exclusively useful in various biomedical applications due to their favorable biological and physical properties [3, 5]. Alginate is a well-known linear anionic biopolymer that consists of α-L-guluronic acid and β-D-mannuronic acid units in different proportions and successive arrangements. The polymer shows interesting properties such as biocompatibility, immunogenicity, biodegradability, nontoxicity, good film-forming, hemostatic potential, desirable mechanical properties, and capacity for bioresorption of the constituent materials that have been widely used for wound dressing application [6, 7]. When water-insoluble calcium alginate is put in contact with wound exudates, calcium ions are released as a result of the replacement of calcium ions with the sodium ions in body fluids that can act as a hemostatic agent. Then, sodium alginate fiber absorbs a large amount of exudates and turns itself into a gel, which keeps the moist interface on the surface of the wound [8, 9].

The ideal wound dressing should protect the wound against microorganism penetration [10, 11]. For wound and burn dressing, various types of antibacterial materials such as chitosan [12, 13], silver nanoparticles (AgNPs) [14, 15], and poly (hexamethylene) biguanide (PHMB) [16] have been used. AgNPs are the most effective against many species of bacteria such as *Staphylococcus aureus* and *Escherichia coli* [17, 18]. The antibacterial mechanism of AgNPs has not been exactly determined; nanoparticles adhere to the bacterial cell membrane, diffuse into the cell, and finally prevent the respiration process [19, 20]. AgNPs have very low toxicity to human cells so they can be used in medical applications. In the case of the wound and burn dressing application, it is very important to use the optimal and the least amount of these particles [21].

Another antibacterial agent is PHMB that has a strong antiseptic effect against gram-negative and gram-positive bacteria with low toxicity and excellent tolerance that has been widely used in the medical industry [22, 23]. PHMB can be used for wound and burn dressing, medical implants, cosmetics, textiles/fibers, and the food industry due to its low-risk profile, chemical stability, reasonable cost, and favorable antibacterial activity [21, 24]. Ashraf et al. showed that the combination of AgNPs and PHMB has about 100 times higher antibacterial properties compared to gram-negative bacteria (*E. coli*) [25].

Recently the development of new wound dressing has attracted lots of attention. One of the most widely used types of wound dressing is cotton gauze. In this article, an appropriate alternative for this type of wound dressing is introduced. On the other hand, there is no investigation of the inhibitory effect of the combination of PHMB and AgNPs onto wound dressing. It seems that combining these two antibacterial materials can improve antimicrobial properties. However, because of the toxicity of antibacterial materials for mammalian cells, the lowest and optimal amount of them should be determined. For this purpose, various compositions of Alginate/PHMB/AgNPs nanocomposite are applied on cotton gauze and polyamide (PA) substrate using the dipping technique. Then, their antibacterial properties, water uptake capacity, and hemostatic properties are compared by considering PHMB and AgNPs interaction.

## Experimental

### Materials

Sodium alginate (medium viscosity) in powder form was purchased from Sigma Aldrich Company. The colloid of AgNPs (4000 ppm) was obtained from Baran Shimi Company, Tehran, Iran (particle size: 10 nm). PHMB (as a 20% w/v aqueous solution) was purchased by Lonza Ltd., Basel, Switzerland. All other chemicals, calcium chloride (99.5%), sodium dihydrogen phosphate, potassium dihydrogen phosphate, sodium hydroxide, potassium chloride, Nutrient broth, and nutrient agar were supplied from Merck Company, Germany. All laboratory chemicals were used without any purification and were of analytical grade. Sterilized gauze (100% cotton) used in the experiments was obtained commercially from Green gauze and Bandage Company. PA substrates (15 Weft in each cm) were used in this study. All solutions were prepared with deionized water and all glasswares were cleaned with water, then dried in an oven. For the antibacterial studies, The *S. aureus* bacteria (gram-positive) with the number of PTCC 1431 was obtained from the Culture Collection Centre (Iranian Research Organization for Science and Technology (IROST)).

### Testing and analysis

The Fourier transform infrared spectroscopy (FTIR) spectra were recorded at room temperature on a Nicolet Magna 560 using KBr pellets (Nicolet, America). Treated cotton gauzes and PA substrates with Alginate, AgNPs, and PHMB were characterized using scanning electron microscopy (SEM) images (Philips XL30i, Netherlands). The samples were coated with a thin layer of gold before SEM. The average particle size and their distributions were determined using Digimizer image analysis software.

### Preparation of PA substrates

For removing the impurities, PA substrates were cleaned with nonionic washing agents and sodium carbonate (0.5%) at a liquor-to-good ratio of 40:1, 70 °C for 10 min, and followed with warm and cold water rinsing for 3 min. The substrates were then dried at ambient temperature. To create new functional groups on PA substrates for improving the hydrophilicity of PA substrates (to simulate most of it with sterile gauze), washed samples were cut in a certain size and treated under various pressure (1, 2, 3, and 4 Torr) and time (2 and 4 min) using plasma treatment (Femto Science Inc, cute model) under oxygen.

### Preparation of alginate–AgNPs and alginate–AgNPs–PHMB composite

In order to prepare an aqueous phase consisting of 0.5% w/v sodium alginate, 0.5 g of alginate powder was dissolved in deionized water at room temperature. Then, the alginate solution was maintained at 3 °C, 24 h for removing the air bubbles. Various concentrations of the AgNPs (25, 50, 75, 100, 150, 200, 225, and 250 ppm) were added to the alginate solution and thoroughly mixed by continuous agitation in a magnetic stirrer to ensure complete dissolution of the polymers. In the next step, the preparation of alginate/AgNPs/PHMB nanocomposite was achieved by mixing various concentrations of the AgNPs (25, 50, 75, and 100 ppm) and PHMB (0.2, 0.4, 0.6, 0.8, and 1% v/v) with alginate solution (0.5% w/v) at room temperature with continuous agitation in a stirrer until a homogeneous suspension was formed.

### Treating of nanocomposites over the gauze and PA substrate

Nanocomposites were deposited over the gauze and the PA substrate by dipping technique. To do this, cotton gauzes and PA substrate were cut into small pieces (0.2 g), in the first step, Samples immersed in the sodium alginate–AgNPs solution, and in the second step, Samples immersed in the sodium alginate–AgNPs–PHMB solutions for about 30 min. Thereafter, the samples were taken out and dipped into calcium chloride (2% w/v) solution, remained undisturbed for 30 min to cross-link with Ca^2+^ forming calcium alginate and ensure the deposition of calcium. Then, the treated samples were dried in an oven (75 °C) for 15 min.

### Water uptake capacity

The water absorption efficiency of wound dressing was evaluated in a phosphate buffer solution (PBS) (simulated body fluid). For evaluating the water uptake capacity, the PBS was made according to Table 1 and buffered at pH 7.4 with sodium hydroxide.

**Table 1:** Ingredients of phosphate buffer solution

Materials	Amount (g)
KCl	0.2
NaCl	8
Potassium dihydrogen phosphate	0.24
Sodium dihydrogen phosphate	1.44

The dry samples were cut into small pieces, weighed, and placed into PBS (pH 7.4) in an incubator at 37 °C for 24 h. For removing surface water, the dressings were removed and put on absorbent paper for a certain time and re-weighed to determine the wet weight. The test was repeated three times for every specimen under the aseptic conditions. The water uptake capability (WUR) of each sample was calculated through the following Eq. (1):

l $$\% {\mathrm{WUR}} = \left( {W - W_0} \right)/W_0 \ast 100$$

Where *W*_0_ is the dry weight of the samples and *W* is the weight of the sample immersed in PBS for 24 h [26].

### Evaluation of antibacterial activity

The antibacterial efficacy of dressing containing various concentrations of AgNPs and PHMB against human pathogenic *S. aureus* was examined by inhibition zone technique (disk diffusion technique). The pure sample (blank gauze and PA substrate) was also determined as a control sample. Certain concentrations of gram-positive bacteria were grown in 10 ml of broth media and then were incubated at 37 °C for 6–8 h. Nutrient agar culture was prepared and spread onto agar plates. Treated and untreated samples were cut into small circular pieces (15 mm diameter) and placed on the surface of the agar plate in separate positions. Next, the agar plates were kept in an incubator at 36 ± 1 °C and after 24 h the inhibition zones were measured in millimeters. The average zone of inhibition around each sample was calculated by using Eq. (2).

2 $${\mathrm{W}} = \frac{{T - D}}{2}$$

Where *W* is the width of the inhibition zone in mm, *T* is the total diameter of treated samples and clear zone in mm, and *D* is the diameter of the treated sample in mm [18].

Furthermore, antibacterial properties of AgNPs on gauze and PA substrate were predicted by using the Matlab program (one-dimensional Interpolation process). Then actual data (experimental data) and the predicted results were compared.

### Evaluation of hemostatic property

In order to investigate whether alginate can increase the rate of blood clotting, human blood was contacted with dressings for a few minutes. As the human blood started flowing out from the finger, a small piece of treated samples was used to absorb the blood immediately. After spending a few seconds, the color change on the gauze and PA substrate was observed. The test procedure was repeated two times for each sample.

## Results and discussion

### Plasma treated on PA substrate

The plasma is an ionized gas environment (electrons, free radicals, ions, metastables, UV-radiations, and photons) that these particles directly or indirectly take part in chemical reactions and cause the surface modification of the fiber without affecting the substrate bulk. Plasma creates new functional groups on the fiber surface or active groups on the fiber surface; and making fibers with more hydrophilic properties [27, 28]. In this study, the plasma treatment was used for PA substrate surface activation and improves the hydrophilic property. Generally, PA substrates are hydrophobic and have low surface energy, so a Contact Angle measurement is used to determine the hydrophilicity properties of treated and untreated PA substrates. The contact angle of the untreated PA substrate was about 78°. However, contact angles decreased to zero after oxygen plasma treatment, showing that the substrates became more hydrophilic. A Significant increase in substrate hydrophilicity and wettability properties can be due to the formation of new polar functional groups such as –COOH and –OH during the plasma treatment.

The optimum time and pressure for plasma treatment were determined using time measurements of water absorption by the PA substrate (Table 2). The Water absorption time of gauze was very fast (about 40 hundredths of a second) and that of the untreated PA substrate was about 20 s. As shown in Table 2, the optimum time and pressure were obtained 4 min and 4 torr, respectively.

**Table 2:** Time and pressure conditions for plasma treatment

Time (min)	Pressure (torr)	Water absorption time (s)
2	1	1.34
4	1	1.47
2	2	1.20
4	2	1
2	3	0.82
4	3	0.74
2	4	0.64
4	4	Very fast

### FTIR analysis of the untreated and treated samples

**Fig. 1:**
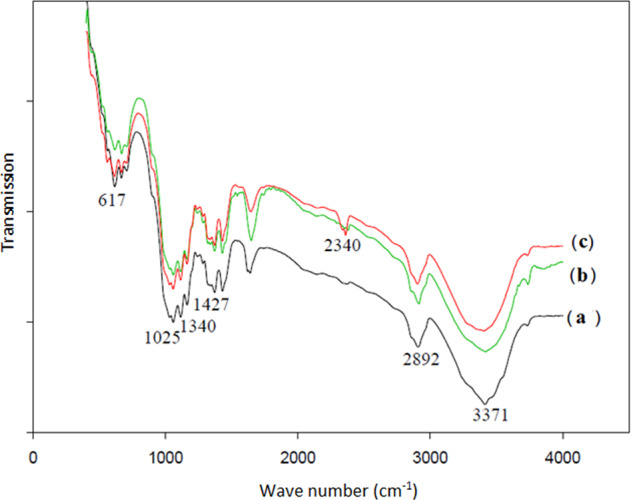
FTIR spectra of (**a**) untreated, (**b**) alginate–AgNPs treated gauzes, and (**c**) alginate–AgNPs–PHMB treated gauzes

**Fig. 2:**
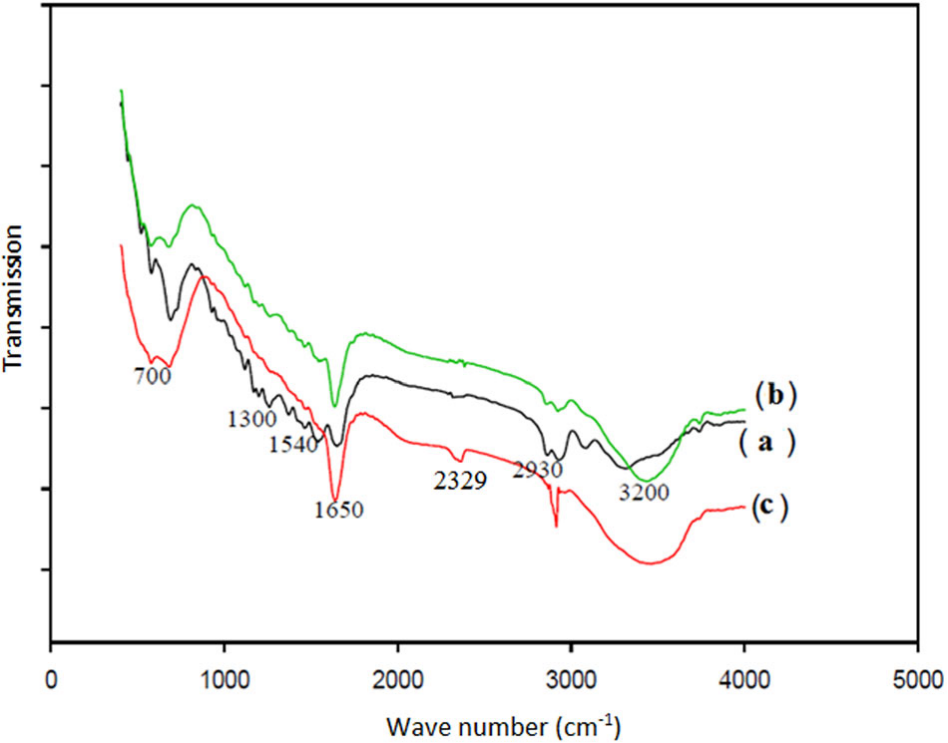
FTIR spectra of (**a**) untreated, (**b**) alginate–AgNPs treated PA fabrics, and (**c**) alginate–AgNPs–PHMB treated PA fabrics

Untreated and treated samples were characterized by FTIR spectrophotometer. For FTIR analysis samples cut finely and mixed thoroughly with about a little amount of KBr, and then spectra were taken in the spectral range of 400–4000 cm^−1^. FTIR spectra of the treated and untreated samples are shown in Figs. 1 and 2. The characteristic peaks of untreated gauze appear at 1025 cm^−1^ (C–O stretching), 1340 cm^−1^ (C–H bending), 1427 cm^−1^ (C–H wagging), 1641 cm^−1^ (carboxylate stretching), 2892 cm^−1^ (C–H stretching), and 3371 cm^−1^ (O–H stretching). Alginate polymer was treated on PA substrate and gauze so the characteristic peak of the carboxylate group appears with stronger intensity (1640–1650 cm^−1^). The characteristic peak for PHMB on gauze and PA substrate is observed at 2340 cm^−1^ and 2329 cm^−1^, respectively, because the main characteristic peaks of PHMB due to nitrogen-related vibrations were located at 2000–2400 nm range. According to Figs. 1 and 2, the spectra of deposited samples do not indicate new peaks different from those of untreated samples, which means that during the coating of nanocomposites on gauze and PA substrate no chemical reaction takes place. In other words, the deposition of composites on the substrates is only the result of physical interactions.

### SEM micrographs

**Fig. 3:**
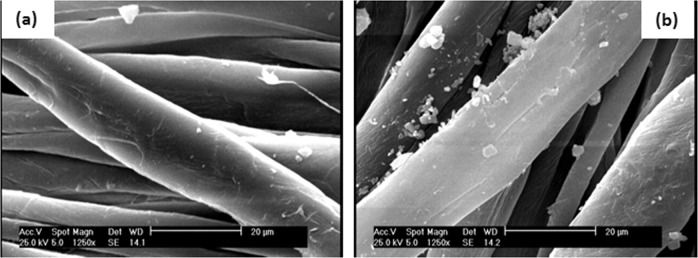
SEM images of (**a**) untreated and (**b**) treated gauze with alginate/AgNPs/PHMB nanocomposite

**Fig. 4:**
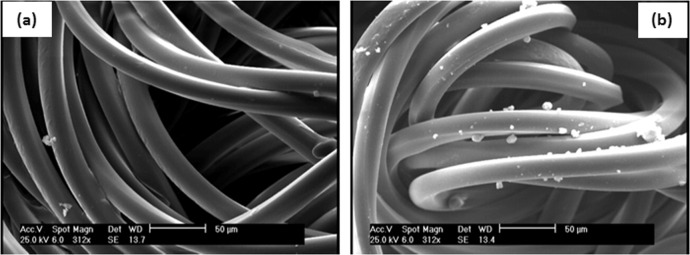
SEM images of (**a**) untreated and (**b**) treated PA fabric with alginate/AgNPs/PHMB nanocomposite

The surface morphology of untreated and treated samples is demonstrated in Figs. 3 and 4. The deposited alginate–AgNPs–PHMB nanocomposite was found on gauze and PA substrate as shown in the SEM microphotographs. It can be noticed that untreated samples have a smooth and homogenous surface, whereas on the treated samples the smooth surface is converted to the rough surface with AgNPs and PHMB, which can be due to the deposition of alginate–AgNPs–PHMB onto the surface of the hospital gauze and PA substrate.

The average diameter of particle size was measured 78 nm on gauze and 172 nm on PA substrate surface (based on 40 nanoparticles).

### Water uptake capacity

Samples (untreated and treated) were immersed in a buffer solution (pH 7.4) and kept at 37 °C for 24 h, thereafter PBS absorption was measured several times. The average data of wound dressing was shown in Table 3. Ideal wound dressing should absorb excess exudate around the wound. According to the results obtained by water uptake capacity, it was specified that the absorption rate of buffer solution is significantly increased on both deposited substrates (gauze and PA substrate) because of the presence of calcium alginate polymer. Alginate polymer has lots of carboxyl and hydroxyl groups and a porous structure resulting in a high capability for absorbing water/body fluids; therefore, the water absorption efficiency of the deposited wound dressing is higher than the untreated one. The water uptake capacity of PA substrate, which is treated with plasma, is more than that of untreated one, and it shows the advantage of applying plasma treatment for PA substrate.

**Table 3:** Water uptake capacity of treated and untreated samples

	Gauze		PA substrate		PA substrate with plasma treatment	
	Untreated	Treated	Untreated	Treated	Untreated	Treated
%WUR	819.64 ± 3.80	1050.85 ± 9.31	500.7 ± 9.19	549.04 ± 9.40	545.64 ± 6.44	638.55 ± 8.91

### Antibacterial activity

**Fig. 5:**
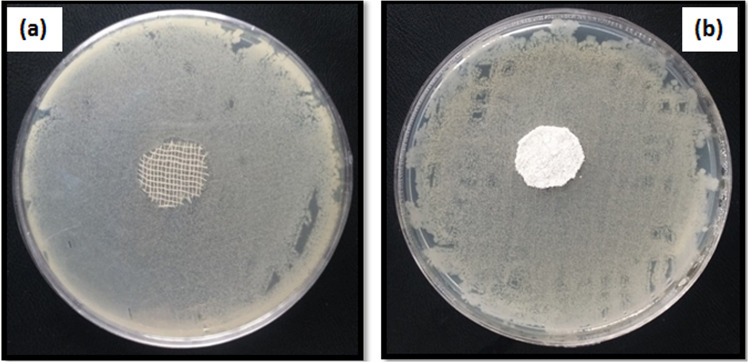
Photograph of the inhibition zone of untreated (**a**) gauze and (**b**) PA fabric against *Staphylococcus* bacteria

**Fig. 6:**
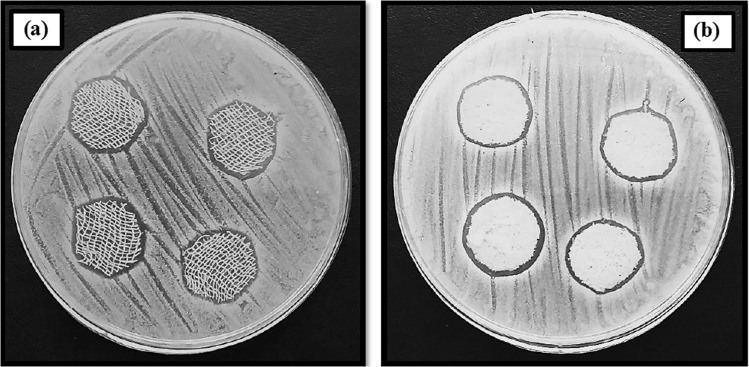
Photograph of the inhibition zone of alginate/AgNPs (**a**) gauze and (**b**) PA fabric against *Staphylococcus* bacteria

**Fig. 7:**
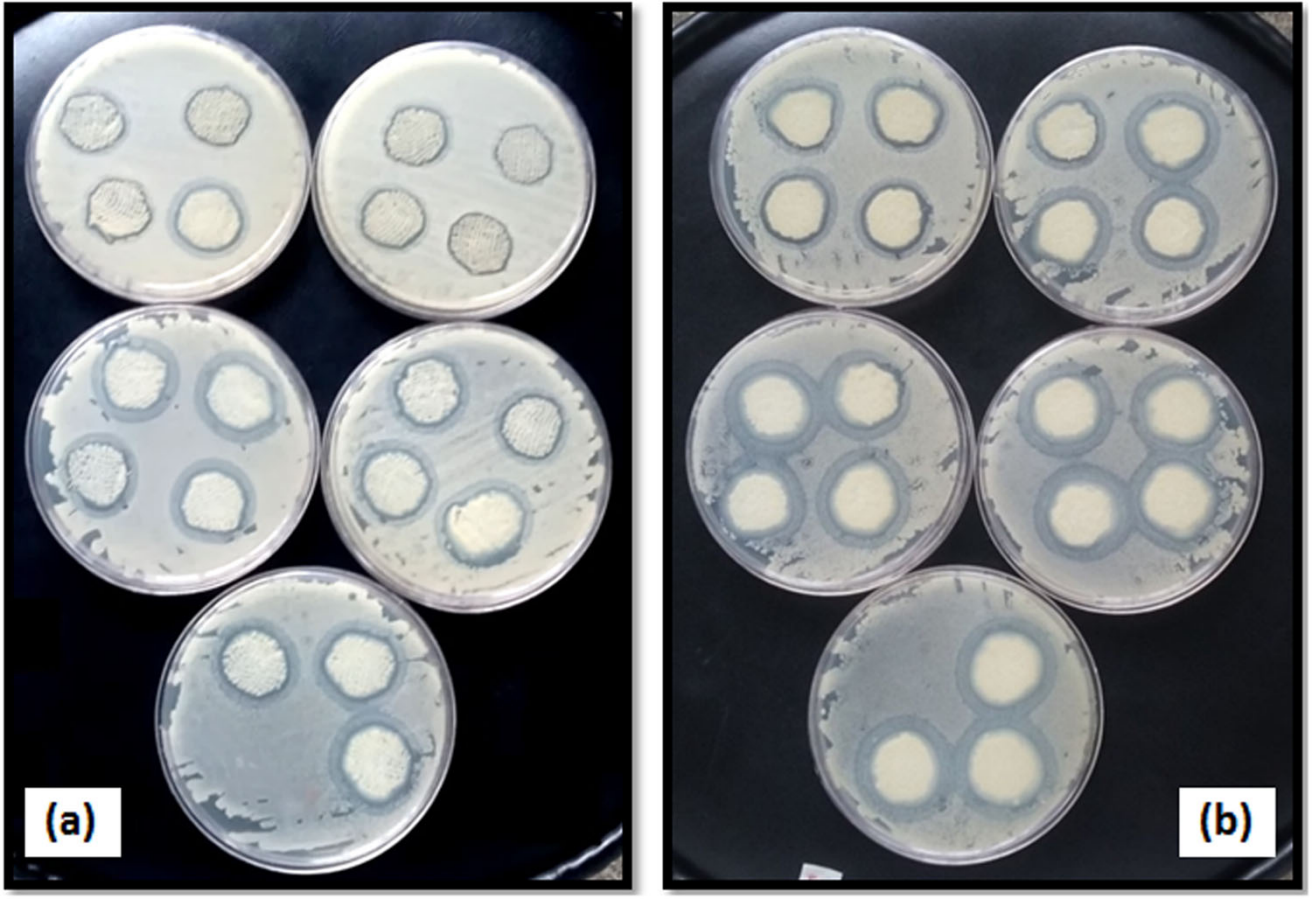
Photograph of the inhibition zone of alginate/AgNPs/PHMB (**a**) gauze and (**b**) PA fabric against *Staphylococcus* bacteria

Wounds are usually exposed to bacterial penetration. In this study, *S. aureus* bacteria were selected for the antibacterial test because it is usually associated with infections during the wound healing process. The antibacterial activity of prepared alginate–AgNPs composite and alginate– AgNPs–PHMB nanocomposite was evaluated by the disc diffusion method on sterile gauze and PA substrate. The prepared samples were placed on agar media and after 24 h incubation; the antibacterial activity of them was measured by the inhibition zone around the samples. Images of the in vitro antibacterial activity of samples are shown in Figs. 5–7. When samples were placed on agar media, antibacterial agents (AgNPs and PHMB) transferred from gauze and PA substrate into the surrounding. So, organisms would not grow around treated samples. As seen in Fig. 5, no clear zone was observed for control samples, implying that untreated samples do not have any antibacterial activity. According to Figs. 6 and 7, there is a clear inhibition zone in each agar plate around the deposited samples, so microorganisms would not grow around treated samples.

**Fig. 8:**
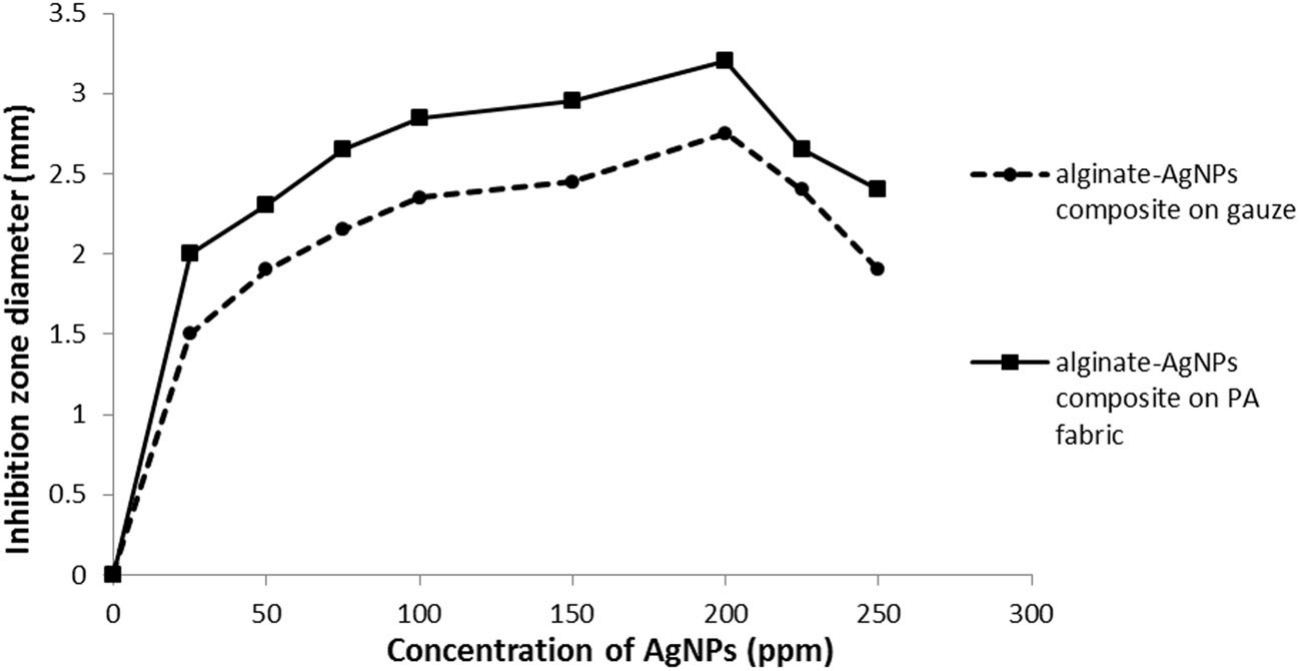
Average inhibition zones diameters of alginate–AgNPs composite on gauze and PA substrate

Figure 8 shows the effect of variations in the AgNPs concentration on the inhibition zone diameter. The size and solubility of AgNPs determine the area of silver infiltration around the gauze and PA substrate. It was observed that by increasing the concentration of AgNPs up to 200 ppm, the growth of the inhibition zone was increased. In fact, the activity of *S. aureus* bacteria was reduced by increasing AgNPs concentration up to a certain possible value. This can be explained as follows: when the concentration of AgNPs is lower than a certain value, these nanoparticles could be adsorbed to negative sites of substrates easily so the antibacterial activity increased. However, in a higher concentration of AgNPs (>200 ppm) the negative sites of substrates are filled and the size of nanoparticles on the gauze and PA substrate increases which causes these nanoparticles to accumulate, therefore the antibacterial activity in concentrations higher than 200 ppm decreases.

**Fig. 9:**
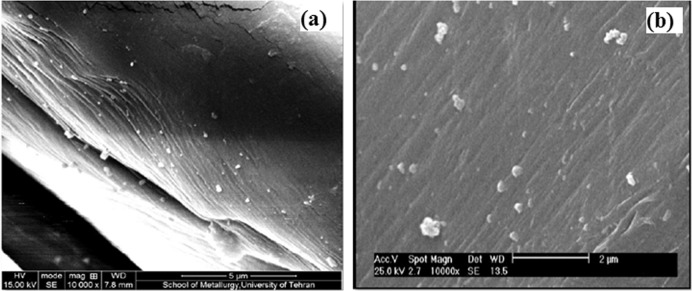
SEM images of treated (**a**) gauze and (**b**) PA fabric with 250 ppm of AgNPs

As mentioned before, the reduction of the antibacterial activity of AgNPs at a concentration above 200 ppm can be due to the accumulation of these nanoparticles. So, SEM was taken from samples with concentrations of 250 ppm to confirm this phenomenon. As seen from Fig. 9, it can be concluded that by increasing the concentration of AgNPs the size of nanoparticles also increases, so the reduction of antimicrobial activity on higher concentrations can be due to the accumulation.

Although the antibacterial activity of treated gauze with AgNPs concentration up to 200 ppm is higher than the others, AgNPs concentrations lower than 200 ppm should be used to protect human health. Since sterile gauze as a wound dressing is in direct contact with human skin, the concentration of AgNPs as an antibacterial agent should be, low in order to prevent the transfer of toxicity to human skin [29, 30]. Therefore, an optimum concentration of AgNPs is 100 ppm which creates favorable antibacterial activity with no toxicity. So, the alginate–AgNPs composite exhibited good antibacterial activity.

Table 4 shows the actual and predicted value (obtained from the one-dimensional Interpolation process) of the antibacterial property of AgNPs on gauze and PA substrate. This table demonstrates a reliable agreement between the actual data (experimental data) and the predicted results.

**Table 4:** Interpolation results of antibacterial property of AgNPs on gauze and PA substrate

		Gauze					PA substrate				
AgNPs		25	75	125	175	225	25	75	125	175	225
Actual value		1.5	2.15	2.25	2.5	2.4	2	2.65	2.95	2.85	2.65
Predicted value											
Linear		0.95	2.12	2.40	2.60	2.32	1.15	2.57	2.77	2.95	2.89
spline		1.20	2.23	2.38	2.63	2.58	1.41	2.74	2.74	2.92	3.19
cubic		1.18	2.19	2.40	2.61	2.50	1.43	2.68	2.77	2.95	3.03
Nearest		1.0	2.35	2.45	2.75	1.9	2.3	2.85	2.7	3.2	2.58
pchip		1.18	2.19	2.40	2.62	2.50	1.43	2.68	2.77	2.95	3.03

**Fig. 10:**
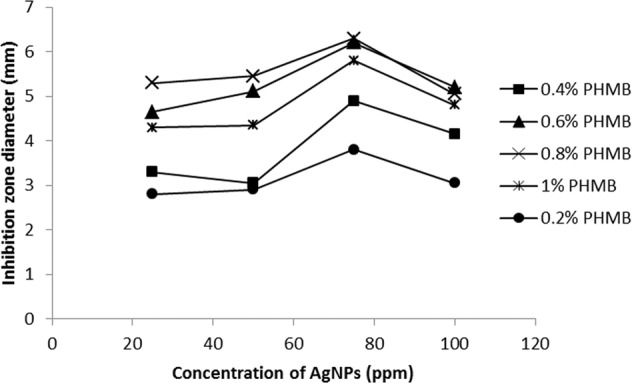
Antibacterial activities of gauzes treated with 0.2, 0.4, 0.6, 0.8, and 1% of PHMB and various concentrations of AgNPs

**Fig. 11:**
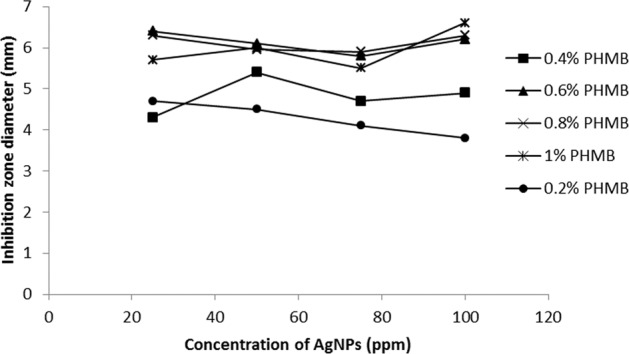
Antibacterial activity of PA substrates treated with 0.2, 0.4, 0.6, 0.8, and 1% of PHMB and various concentrations of AgNPs

The average diameters of zone of inhibition for the sample containing various concentrations of AgNPs and PHMB were measured and the results are shown in Figs. 10 and 11. In Fig. 10, at any concentration of PHMB by increasing AgNPs loadings in the substrates, the inhibition zones increase rapidly first and then decrease. Actually, there is an optimal amount of PHMB and AgNPs for controlling bactericidal efficacy, so the optimal amount of polyhexamethylene biguanide can be selected equal to 0.6% for both substrate and an optimal amount of AgNPs on gauze substrate is 75 ppm and on PA substrate is about 50 ppm. The results demonstrated that using small amounts of both antibacterial agents can obtain desirable antibacterial properties on gauze and PA substrates as a wound dressing. According to Figs. 10 and 11, in each concentration of antibacterial materials (PHMB and AgNPs) the inhibition zones on PA substrate are more than those of gauze.

### Hemostatic properties

**Fig. 12:**
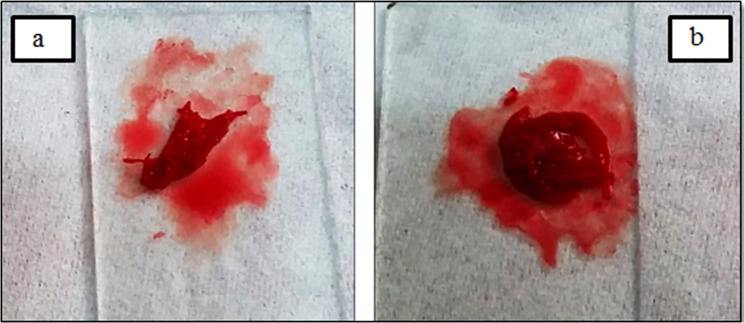
Photograph of hemostatic properties (**a**) untreated and (**b**) treated gauze

**Fig. 13:**
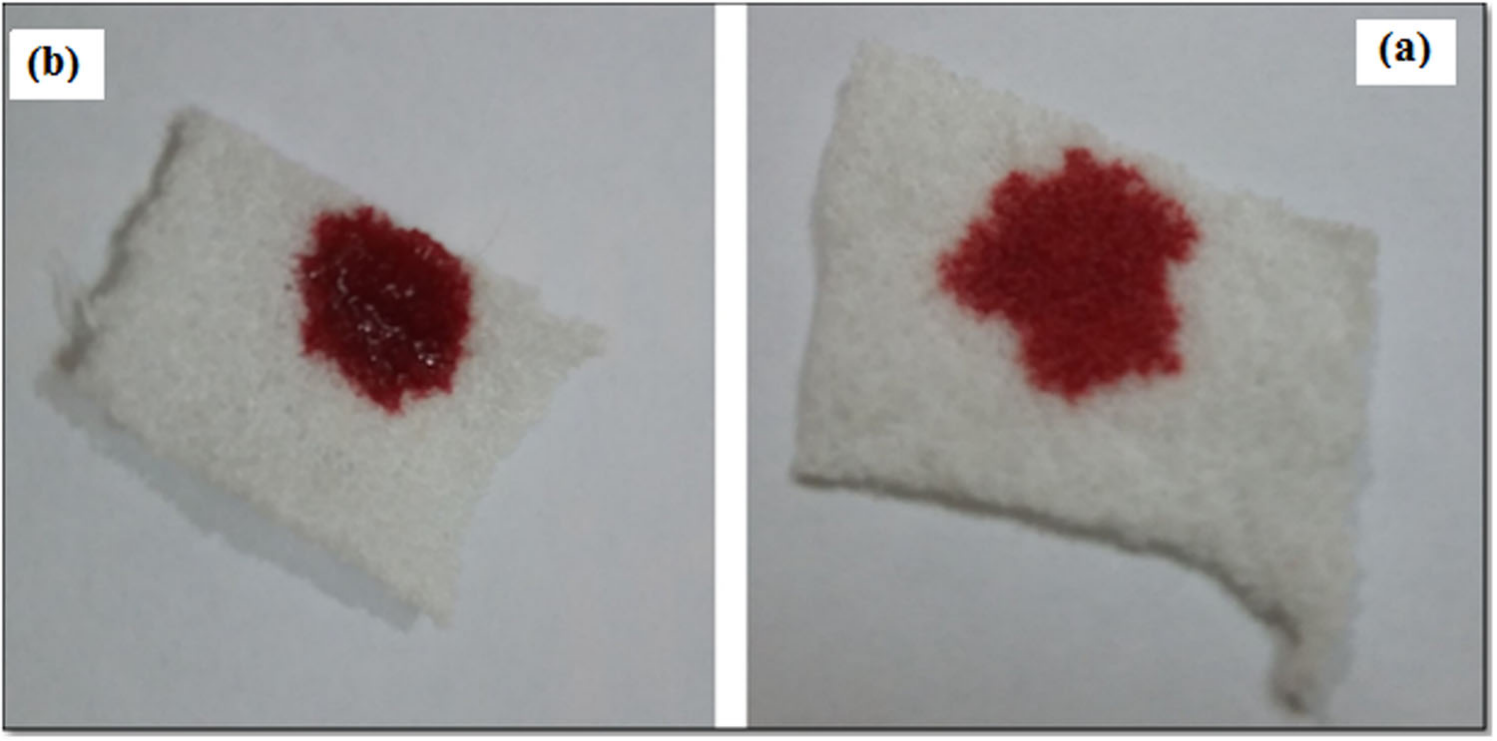
Photograph of hemostatic properties (**a**) untreated and (**b**) treated PA fabric

The hemostatic effect of alginate treated samples was evaluated by healthy human blood. The results of the hemostatic test demonstrated that the attachment of calcium alginate to the surface of the gauze and PA substrate improved the hemostatic property. Actually, whole blood clotting time was significantly reduced when blood contacted with nanocomposite treated samples. Figures 12 and 13 show more rapid clot formation on treated samples compared to the untreated ones. This result could be due to the replacement of calcium ions in nanocomposite with sodium ions in body fluid resulting in free calcium ions.

A higher absorbance rate of the human blood on a substrate shows a slower clotting rate. This phenomenon has been postulated using the remaining applied blood on the surface of the samples. Treated samples with 0.5% (w/v) alginate caused significantly lower absorbance rates than untreated samples (Figs. 12 and 13). This demonstrated that gauze and PA substrate containing alginate was able to cause considerable blood clotting. As Figs. 12 and 13b show when a certain amount of blood contact with the surface of the alginate treated samples, blood immediately turned dark red and gradually caused clots on the wound dressing. However, according to Figs. 12 and 13a, the blank samples absorbed the blood completely. Therefore, alginate is an effective hemostat polymer that can be used in wound and burn dressing.

## Conclusions

Our attempt in this research is to present the PA substrate, a suitable substitution for cotton gauze as a wound dressing. To reach this target, at first we make hydroxyl groups with oxygen gas plasma operations on the PA substrate surface, which increases its hydrophilicity and is able to make the PA substrate similar to cotton gauze in absorbing water. Then, all the operations done to the cotton gauze substrate were similarly repeated on the PA substrate; by FTIR and SEM test it is shown that nanocomposite is properly deposited into the PA substrate just like the cotton gauze. Investigating the information derived from antibacterial experiments, we conclude that the antibacterial property gained from the PA substrate is considerably more than the cotton gauze; which is a positive point to replace the PA substrate to dress wounds. Calcium alginate, which provides a moist environment around the wound, can improve the wound healing process and AgNPs and PHMB create favorable antibacterial activity on hospital gauze and PA substrate. The combination of the mentioned essential properties could obviously reduce energy consumption and the costs of production. On the other hand, it increases safety.

The water absorption capacity of the deposited samples was increased significantly and coagulation time decreased due to the presence of alginate polymer. Water uptake capacity of the PA substrate which was treated under plasma operations was notably increased compared with that of the non-plasma treated sample. However, the plasma operation did not increase the PA substrate’s water absorption scale more than that of the cotton gauze; therefore, the PA substrate’s water absorption is acceptable.

On the other hand, in contrast to PA substrate, cotton gauze leaves tiny particles and fibers onto the wound. Also, flexibility is one of the ideal wound dressing features for applying it over the uneven parts of the body and this is the advantage of PA substrate to the cotton gauze. Finally, considering all the results and stated points, it is concluded that the PA substrate produced during the current research could be an appropriate replacement for the cotton gauze as a wound dressing.
